# Metabolic Analysis of Vitreous/Lens and Retina in Wild Type and Retinal Degeneration Mice

**DOI:** 10.3390/ijms22052345

**Published:** 2021-02-26

**Authors:** Elisa Murenu, Sarantos Kostidis, Shibojyoti Lahiri, Anna S. Geserich, Axel Imhof, Martin Giera, Stylianos Michalakis

**Affiliations:** 1 Department of Ophthalmology, Ludwig-Maximilians-Universität München, Mathildenstraße 8, 80336 Munich, Germany; elisa.murenu@cup.lmu.de; 2 Department of Pharmacy, Ludwig-Maximilians Universität München, Butenandtstr. 7, 81377 Munich, Germany; annageserich@gmail.com; 3 Leiden University Medical Center, Center for Proteomics & Metabolomics, P.O. Box 9600, 2300 RC Leiden, The Netherlands; s.kostidis@lumc.nl (S.K.); m.a.giera@lumc.nl (M.G.); 4 Biomedical Center Munich-Molecular Biology, Ludwig-Maximilians-Universität München, Großhaderner Strasse 9, 82152 Planegg-Martinsried, Germany; Shibojyoti.Lahiri@med.uni-muenchen.de (S.L.); imhof@lmu.de (A.I.)

**Keywords:** metabolism, retina, vitreous/lens, physiology, degeneration, eye opening

## Abstract

Photoreceptors are the light-sensing cells of the retina and the major cell type affected in most inherited retinal degenerations. Different metabolic pathways sustain their high energetic demand in physiological conditions, particularly aerobic glycolysis. The principal metabolome of the mature retina has been studied, but only limited information is available on metabolic adaptations in response to key developmental events, such as eye opening. Moreover, dynamic metabolic changes due to retinal degeneration are not well understood. Here, we aimed to explore and map the ocular metabolic dynamics induced by eye opening in healthy (wild type) or *Pde6b*-mutant (retinal degeneration 1, Rd1) mice, in which photoreceptors degenerate shortly after eye opening. To unravel metabolic differences emerging before and after eye opening under physiological and pathophysiological conditions, we performed nuclear magnetic resonance (NMR) spectrosco-py-based metabolome analysis of wild type and Rd1 retina and vitreous/lens. We show that eye opening is accompanied by changes in the concentration of selected metabolites in the retina and by alterations in the vitreous/lens composition only in the retinal degeneration context. As such, we identify N-Acetylaspartate as a potential novel vitreous/lens marker reflecting progressive retinal degeneration. Thus, our data can help elucidating mechanisms underlying key events in retinal physiology and reveal changes occurring in pathology, while highlighting the importance of the vitreous/lens in the characterization of retinal diseases.

## 1. Introduction

Inherited retinal disorders (IRDs) are among the leading causes of retinal dysfunction (https://sph.uth.edu/retnet/disease.htm, 29 accessed on January 2021), with more than 300 mutated genetic loci associated to pathologies [1]. Most mutations affect the rod and/or cone photoreceptors, which account for >80% of all retinal cells and have the highest metabolic consumption within this tissue [2]. As a result of such mutations, the physiological metabolism is either directly or indirectly disrupted, leading or contributing to a degeneration and loss of photoreceptors. Secondary to the degeneration of photoreceptors, other cells that establish physical or functional connections with them, e.g., retinal pigmented epithelium (RPE), Müller glia, microglia, and retinal interneurons [3,4,5,6], are affected and—depending on the cell type—adapt to the primary photoreceptor degeneration, for instance, by activation, proliferation, or degeneration. Thus, the characterization of the metabolic events preceding and accompanying the development of IRDs is likely to reflect progressive degeneration and can be crucial to establish a rapid and effective course of action in the treatment of a disease.

Despite their high energetic demands, photoreceptors base their metabolism mainly on aerobic glycolysis (the so-called Warburg effect), possibly to employ the intermediate metabolites of the reaction for the constant biogenesis of the disks in the outer segment [7,8,9]. The choroidal blood is responsible for the supply of Glucose, which reaches the photoreceptors after crossing the RPE [10]. Fatty acid consumption and oxidative phosphorylation also contribute to photoreceptors metabolism, particularly to sustain ion pump activity in the inner segments [2,11,12]. Oxidative phosphorylation was also reported in the outer segments of rod photoreceptors, as enzymes of the respiratory chain were detected in the disk membranes and proposed to provide a local energy source, possibly to support the visual cascade [11,12,13,14,15,16]. Additionally, alternative metabolic pathways are not only observed in distinct cell compartments, but also in response to different illumination conditions [17,18,19]. For instance, light has been associated to a decrease in oxygen consumption and glycolytic rate in the retina, as well as to a reduction in purines and pyrimidines that takes place mainly in photoreceptors [17]. Surprisingly, however, the effects of light exposure on metabolome coinciding with eye opening are instead still unknown.

Metabolites generated in all these conditions can in turn reflect on the composition of structures in close contact with the retina, such as the lens and the vitreous [20], which are both largely acellular and have little metabolic activity *per se* [21]. As such, few attempts at characterizing the metabolic profile of retina, vitreous and lens have been made [22,23,24,25,26,27], some of which in relation to systemic pathological conditions [26,27]. However, to the best of our knowledge the impact of IRDs on eye metabolism has only marginally been addressed [17,28], and a correlation among metabolomes in retina and vitreous/lens has yet to be investigated.

Here, we perform a metabolic analysis of retina and vitreous/lens in postnatal mice prior to as well as shortly after eye opening to pinpoint physiological mechanisms triggered by this key developmental event. By applying the same analysis to the *Pde6b^Rd1^* (Rd1) mutant mouse model, whose severe and early-onset photoreceptor degeneration is known to be aggravated with eye opening, we show that the metabolic composition of the vitreous/lens is affected over time only when a degenerative context is provided, leading us to identify N-Acetylaspartate as a putative degeneration marker.

We believe the results of this extensive metabolomic analysis in retina and vitreous/lens at key time-points can help unravelling adaptation mechanisms taking place around eye opening in the healthy retina. Moreover, our data can help shed light on metabolic processes associated with disease onset and progression, thus potentially providing new prognostic markers of retinal degeneration.

## 2. Results

### 2.1. More Than Meets The Eye: Metabolic Dysfunctions Underlie Retina Degeneration

**Figure 1:**
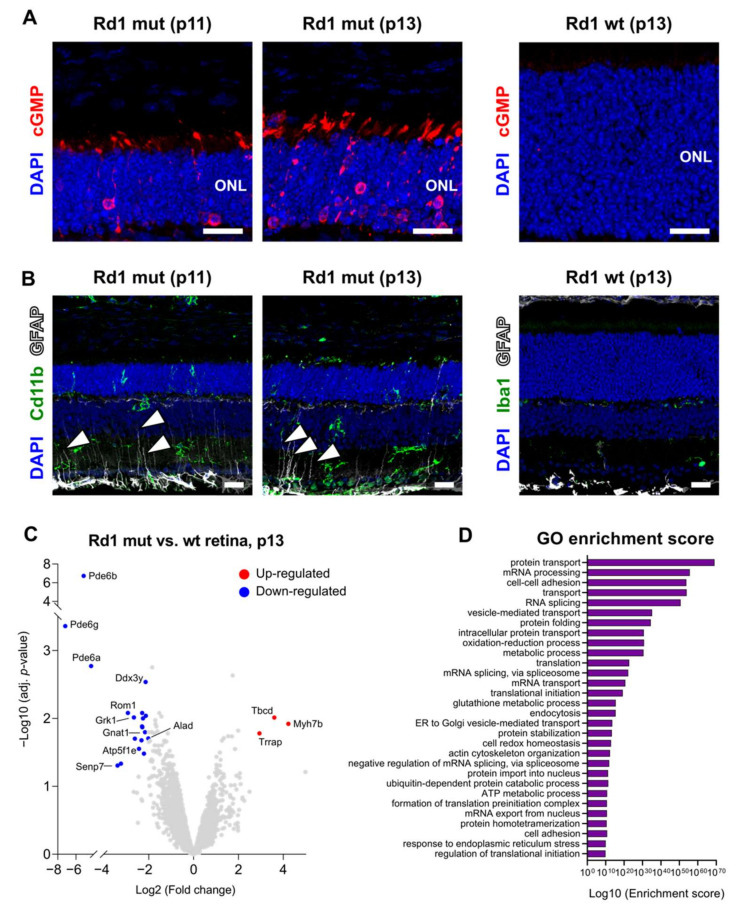
Retinal degeneration in the Rd1 mutant mouse. (**A**) Maximum intensity projection of confocal images showing immunolocalization of cGMP (red) in the outer nuclear layer (ONL) in the Rd1 mutant mouse at p11 and p13 and in the Rd1 wt control at p13. (**B**) Reactivity and distribution of glial cells across different retina layers. Arrowheads indicate the processes of Müller glia cells with a positive GFAP signal. (**C**) Volcano plot of protein expression ratios between Rd1 mutant and Rd1 wt at p13. Dots in blue or red indicate proteins that are significantly down- or up-regulated, respectively. (**D**) Gene Ontology analysis performed on all proteins and showing the 30 terms with the highest enrichment score. Rd1 wt: *n* = 4; Rd1 mutant: *n* = 4. Scale bars: 20 μm.

The Rd1 mutant mouse line is a model of early-onset retinal degeneration that affects primarily rod photoreceptors and shows rapid progression when the animal first opens the eyes at around postnatal day 13 (p13) [3,29,30]. The lack of rod phosphodiesterase function in Rd1 mutants results in the accumulation of the second messenger cyclic guanosine monophosphate (cGMP) in rod photoreceptors and ultimately to the degeneration of these cells by cytotoxicity [31], which highly depends on the rod cGMP-gated cation channel [32]. Indeed, immunolabeling for cGMP revealed an accumulation of this second messenger in Rd1 mutant rod photoreceptors already prior to eye opening (at p11) and was further increased after eye opening (at p13) (Figure 1A). In contrast, cGMP signal remained below the detection level of the antibody in Rd1 wt retinas (Figure 1A). Immunostaining of Rd1 mutant retinas at these early postnatal stages around eye opening also showed elevated levels of various markers of retinal degeneration [29]. This aspect was exemplified by immunolabeling for the astrocytes and Müller glia cell marker glial fibrillary acidic protein (GFAP) and the microglia/monocyte marker cluster of differentiation molecule 11b (Cd11b). Indeed, GFAP and Cd11b indicated a glial response to retinal degeneration in Müller glia processes and the activation of microglia cells, respectively, as opposed to Rd1 wt retinas at p13, which showed no GFAP reactivity in Müller glia processes and only a weak signal of the pan-microglia marker ionized calcium binding adaptor molecule 1 (Iba1) (Figure 1B).

In order to map changes in rod degeneration at eye opening, we subsequently performed a proteomic analysis of Rd1 mutant and wt retinas at p13. Cumulatively, we identified 5322 proteins, among which 16 and 24 proteins were only detected in the Rd1 mutant and wt retinas, respectively, and 5146 were shared (Table S1). Statistical analysis of these shared proteins showed a significant enrichment (>2 fold and *p*-value < 0.05) of 19 proteins in the wt condition, while three proteins were found enriched in Rd1 mutant animals (Figure 1C). As expected, known key proteins of the visual cascade (Pde6a, Pde6b, Rom1, Gnat1, and Grk1) were found among the underrepresented proteins in Rd1 mutant mice (Figure 1C). However, a link to cellular metabolism also emerged from the comparison (Table S1), as indicated by proteins involved in the respiratory chain (Atp5f1e), metabolism-related proteins (Alad), and enzymes implicated in various functions (Ddx3y, Senp7). Of note, gene ontology (GO) analysis of all proteins further underlined the enrichment of terms associated to metabolism (Figure 1D, Table S1).

### 2.2. A Comprehensive Metabolomic Analysis Unravels Differences Across Tissues, Age, Health, and Disease

As suggested by several studies [6,33,34] and by our proteomic analysis, the failure of the physiological metabolism in photoreceptors is likely to participate in their degeneration. However, whether metabolic changes are causative or epiphenomenal is still unclear.

**Figure 2:**
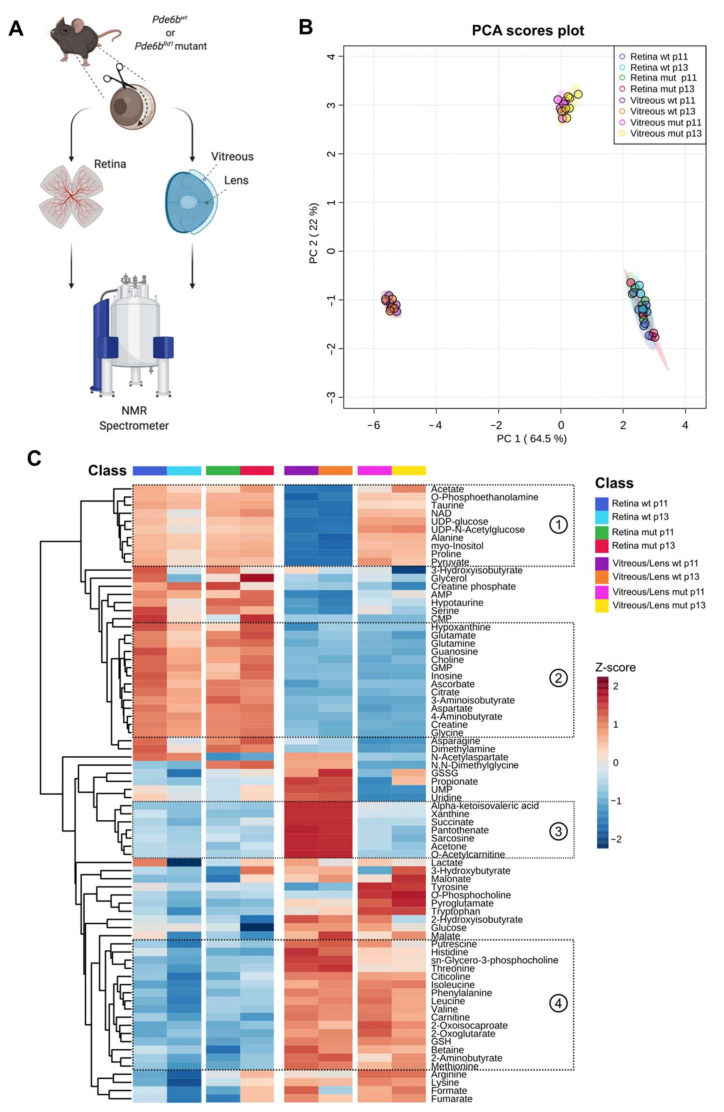
A comprehensive metabolomic analysis reveals time-, compartment-, and degeneration state-dependent differences. (**A**) Schematic overview of the experimental workflow. (**B**) Principal component analysis of all collected metabolomic data, reflecting differences regarding the time-point of analysis (p11, p13), the compartment (retina and vitreous/lens), and the phenotype (wt, Rd1 mutant). (**C**) A heatmap representing the z-score of absolute concentration levels of 76 metabolites across all conditions, clustered according to the Euclidean distance. The circled numbers (1 to 4) indicate the clusters discussed in the main text. Rd1 wt, vitreous/lens: *n* = 6 (p11), *n* = 5 (p13); retina: *n* = 7 (p11), *n* = 4 (p13). Rd1 mutant, vitreous/lens: *n* = 6 (p11), *n* = 7 (p13); retina: *n* = 5 (p11), *n* = 6 (p13).

To elucidate this aspect, we applied NMR spectroscopy to analyze the abundance of metabolites in the retina of Rd1 mutant and wt mice shortly before (p11) and after (p13) eye opening (Figure 2A). Additionally, we opted to analyze the metabolically less active vitreous/lens, aiming to gain insight into metabolites that are secreted by the retina and accumulate in these adjacent ocular structures (Figure 2A). We, therefore, focused our analysis around three variables: the time-point (p11 vs. p13), the degeneration state (wt vs. Rd1 mutant), and the tissue compartment (retina vs. vitreous/lens) (Table S2).

In an attempt to simplify the data and get a first overview, we performed a principal component analysis (PCA) of all metabolomics data (Figure 2B). While overall the retina samples clustered separately from the vitreous/lens, as expected from the diverse nature of the two compartments, the time-point and phenotype components reflected way subtler differences in the retina. Strikingly, the vitreous/lens samples showed a distinct clustering pattern depending on the phenotype, suggesting substantial differences in the metabolite composition of this compartment that are the result of the degenerative context.

Next, we focused on the concentration values of the single metabolites (Figure 2C). As seen in the PCA plot, the vitreous/lens displayed changes that resulted in very discrete clusters based on the genotype (cluster 1,3), while other clusters mainly reflected the biological dissimilarity from the retina (cluster 2,4). Interestingly, only selected metabolites accounted for differences across time-points in both tissues and between phenotypes within the context of the retina.

Taken together, the high-level analysis of the metabolome suggests a higher degree of difference in the vitreous/lens compared to the retina, with the biggest effect being exerted by the phenotype.

### 2.3. Eye Opening Results in Minimal Effects on the Wild Type Retina and Vitreous/Lens

**Figure 3:**
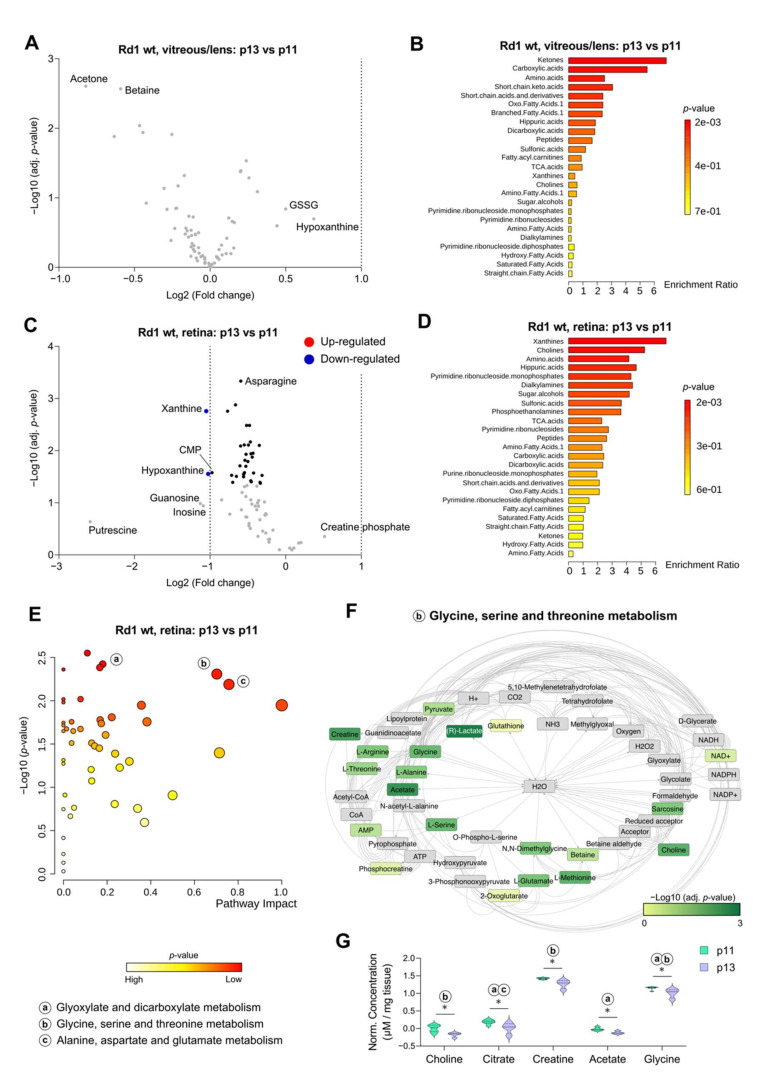
Metabolite analysis in the wt retina reveals distinct differences caused by eye opening. Volcano plot (**A**) and related quantitative metabolite set enrichment analysis (**B**) illustrating changes in the metabolite concentration in the vitreous/lens of Rd1 wt mice between time-points p13 and p11. Volcano plot (**C**) and related quantitative metabolite set enrichment analysis (**D**) illustrating changes in the metabolite concentration in the retina of Rd1 wt mice between time-points p13 and p11. (**E**) Pathway analysis of the retina data depicted in subfigures (**C**,**D**), showing the significance of the time-point comparison in relation to the topology (impact). Small letters indicate the pathways in the legend. (**F**) Cytoscape network representation of pathway “b” selected from subfigure (**E**). The metabolites with a match in the pathway are shown color-coded according to their significance in our analysis. (**G**) Violin plots of metabolites selected in subfigure (**F**) for their significance, showing their concentrations at both time-points. The letters indicate the corresponding pathway from subfigure (**E**) in which the metabolites are found. Vitreous/lens: *n* = 6 (p11), *n* = 5 (p13). Retina: *n* = 7 (p11), *n* = 4 (p13). Multiple unpaired t test with Holm-Šídák correction for multiple comparisons. * *p* < 0.05.

Following this overview analysis of the data, we set out to simplify their interpretation by performing targeted analysis, all the while keeping some variables separated, e.g., the genotype. To establish the metabolic dynamics of healthy retina and vitreous/lens around eye opening, we first focused on the wt data. As suggested by the overview analysis (Figure 2B), no substantial change was detected in the vitreous/lens from p11 to p13 (Figure 3A). Quantitative metabolite set enrichment analysis (MSEA) revealed only a few significant changes in the classes enriched at p13 compared to p11, with Ketones displaying the highest score (Figure 3B). Conversely, in the retina 2 metabolites (Xanthine and Hypoxanthine) were found significantly underrepresented at p13 (Figure 3C) and contributed to the most enriched metabolic class emerging from the time-point comparison (“Xanthines”, Figure 3D). Strikingly, only a small proportion of metabolites (six, accounting for 8% of total) seemed to be upregulated at p13 (Figure 3C), albeit non-significantly, suggesting that eye opening is accompanied by a general metabolic suppression in the retina.

Having observed some significant differences in the retina, we decided to focus on this eye compartment to explore possible changes in metabolic pathways. Indeed, several pathways resulted particularly relevant (both in terms of enrichment and topology) in the comparison between p13 and p11 retina, including “glyoxylate and dicarboxylate metabolism”; “glycine, serine, and threonine metabolism”; and “alanine, aspartate, and glutamate metabolism” (Figure 3E). To confirm these results, and as an example of our pipeline of analysis, we subsequently focused on “glycine, serine, and threonine metabolism”. The underlying network revealed the involvement of several metabolites from our input, thereby supporting the data obtained with the previous analysis (Figure 3F). Taking advantage of the network analysis, we focused on selected metabolites with the lowest *p*-value in the comparison between time-points, namely Choline, Citrate, Creatine, Acetate, and Glycine, aiming to confirm their differential regulation (Figure 3G). Indeed, we found a significant decrease of these metabolites at p13, thus supporting changes taking place in several metabolic pathways between the two time-points.

Altogether, we concluded that in physiological conditions the vitreous/lens metabolome remains largely unvaried, while few changes can be detected in the retina as part of a general metabolic reduction taking place around eye opening.

### 2.4. The Rd1 Mutation Induces Changes in the Composition of Vitreous/Lens at Eye Opening

**Figure 4:**
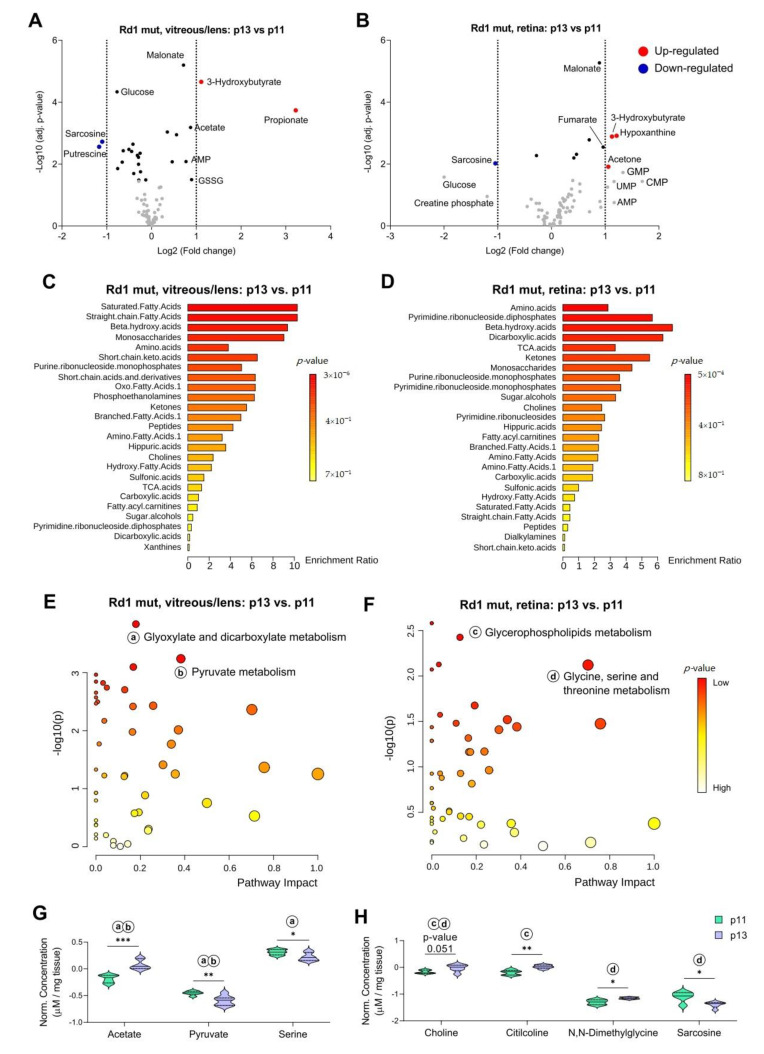
Photoreceptor degeneration affects the metabolite composition of vitreous/lens and retina across time. (**A**,**B**) Volcano plots with the differences between time-point p13 and p11 of metabolites concentration in the vitreous/lens (**A**) and the retina (**B**) of Rd1 mutant mice. (**C**,**D**) Quantitative metabolite set enrichment analysis showing the metabolite classes enriched in vitreous/lens (**C**) and retina (**D**) between the two time-points. (**E**,**F**) Pathway analysis with the significance of the time-point comparison in the vitreous/lens (**E**) and retina (**F**) in relation to the topology (impact). Selected pathways with a high significance are indicated (small letters). (**G**,**H**) Violin plots with summary quantification data of metabolites with a high significance within the indicated pathways in subfigures (**E**,**F**) in the vitreous/lens (**G**) and retina (**H**). The concentrations are showed at both time-points. The letters indicate the pathway in subfigures (**E**,**F**) in which the metabolites are found. Vitreous/lens: *n* = 6 (p11), *n* = 7 (p13). Retina: *n* = 5 (p11), *n* = 6 (p13). Multiple unpaired t test with Holm–Šídák correction for multiple comparisons. * *p* < 0.05, ** *p* < 0.01, *** *p* < 0.001.

After addressing changes in the metabolite composition of wild type retina and vitreous/lens that occur around eye opening, we next asked what differences are induced in those ocular tissues in the degenerative context of the Rd1 mutation. Whereas in wt conditions the vitreous/lens did not show any substantial change (Figure 3A), the Rd1 mutation caused two metabolites to be significantly increased (3-Hydroxybutyrate and Propionate) and another two to be decreased (Sarcosine and Putrescine; Figure 4A). Interestingly, Sarcosine and 3-Hydroxybutyrate were equally regulated in the retina, while the increase in the levels of Hypoxanthine and Acetone was only observed in the vitreous/lens (Figure 4B). Along the same line, different classes of enriched metabolites characterized the passage from p11 to p13 in the Rd1 mutant vitreous/lens and retina (Figure 4C,D) compared to the Rd1 wt condition (Figure 3B,D). As these findings reflected a diverse metabolic composition of the two eye compartments in a context of degeneration, we next investigated their global impact on known metabolic pathways. Vitreous/lens and retina showed a different pattern of enrichment of metabolic pathways, with “glyoxylate and dicarboxylate metabolism” and “pyruvate metabolism” being more important in the first compartment and “glycerophospholipids metabolism” and “glycine, serine, and threonine metabolism” in the second (Figure 4E,F). Of note, “glycerophospholipids metabolism” emerged in the Rd1 mutant context, but not in the Rd1 wt (Figure 3E), suggesting degeneration-specific alterations associated with this pathway. The most prominently altered metabolites, namely, Acetate, Pyruvate, and Serine, for the vitreous/lens and Choline, Citilcoline, N,N-Dimethylglycine, and Sarcosine for the retina, further confirmed the differences observed in the Rd1 context at p13 compared to p11 (Figure 4G,H).

Thus, our data indicate that in the retinal degeneration context, selected metabolites are differentially represented at p13 compared to p11. Importantly, only in Rd1 mutant mice the vitreous/lens shows significant changes in its metabolite composition around eye opening. Furthermore, specific metabolites undergo the same changes also in the retina, suggesting an initial equilibration of the metabolic content of these two compartments and the loss of the physiological separation between them.

### 2.5. Differences in Selected Metabolites Underlie The Impact of Degeneration on Vitreous/Lens and Retina Across Time-Points

**Figure 5:**
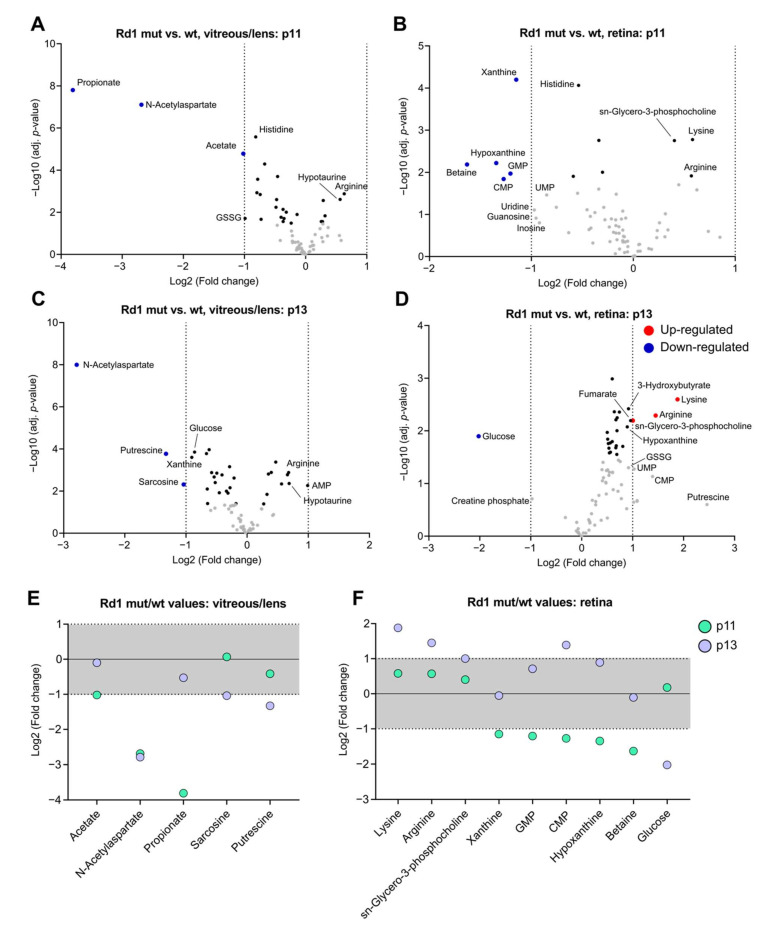
Photoreceptor degeneration results in postnatal stage-dependent deregulation of selected metabolites in vitreous/lens and retina. (**A**–**D**) Volcano plots showing the changes in metabolite concentration between Rd1 mutant and wt in the vitreous/lens (**A**,**C**) and the retina (**B,D**) at p11 (**A,B**) and p13 (**C**,**D**). (**E**,**F**) Summary graphs showing the concentration differences between Rd1 mutant and wt in vitreous/lens (**E**) and retina (**F**) of the significantly deregulated metabolites in (**A**–**D**). The gray area delimits the cut-off of the Log2 (Fold change). Rd1 wt, vitreous/lens: *n* = 6 (p11), *n* = 5 (p13); retina: *n* = 7 (p11), *n* = 4 (p13). Rd1 mutant, vitreous/lens: *n* = 6 (p11), *n* = 7 (p13); retina: *n* = 5 (p11), *n* = 6 (p13).

In order to dissect changes in the metabolic composition in response to photoreceptor degeneration, we next set out to compare Rd1 mutant and wt conditions, while keeping the other variables independent. Compared to wt, the vitreous/lens samples of Rd1 mutant mice showed a significant reduction in the concentration of few metabolites at p11 (Propionate, N-Acetylaspartate and Acetate), of which N-Acetylaspartate remained downregulated at p13. Moreover, the levels of Putrescine and Sarcosine were significantly altered at p13 only (Figure 5A,C,E). Notably, at p13 we also found a less pronounced (close to the fold change cut-off) downregulation of Glucose and Xanthine and upregulation of Adenosine monophosphate (AMP) (Figure 5C). As such, these changes in the vitreous/lens metabolic composition might reflect major energetic imbalances caused by the retinal degeneration in Rd1 mutant mice.

Subsequently, we focused on the analysis of degeneration-specific metabolic changes in the retina samples. Prior to eye opening, the degenerative context of the Rd1 mutant mouse determined a significant decrease in the levels of Xanthine and Hypoxanthine, both involved in purine catabolism (Figure 5B,F). Similarly, the levels of Betaine and specific nucleotide monophosphates, namely, Cytidine monophosphate (CMP) and GMP, was found significantly decreased (Figure 5B,F). Of note, the decrease in GMP levels is in line with the lack of a functional Pde6b enzyme in rods, which ultimately determines the accumulation of cGMP in these cells (Figure 1A). Interestingly, the overall retina metabolome at p13 shifted drastically towards generally increased metabolite concentrations (Figure 5D). Consequently, Lysine, Arginine, and sn-Glycero-3-phosphocholine, which were already partially increased at p11 (Figure 5B), were significantly overrepresented (Figure 5D,F), with the elevated levels of sn-Glycero-3-phosphocholine possibly reflecting a damage in the cell membrane of degenerating rods or an impairment in the phagocytosis of outer segment disks that peaks at eye opening [35,36,37,38]. Conversely, Glucose was the only metabolite whose levels were significantly decreased around eye opening (Figure 5D,F), in agreement with previous findings [39] and similarly to the decrease observed in the vitreous/lens (Figure 5C).

Thus, we concluded that the vitreous/lens partly reflects the metabolic changes seen in the retina, where an initial decrease in purine metabolism and the subsequent decline in Glucose levels suggest an energy imbalance during degeneration.

**Figure 6:**
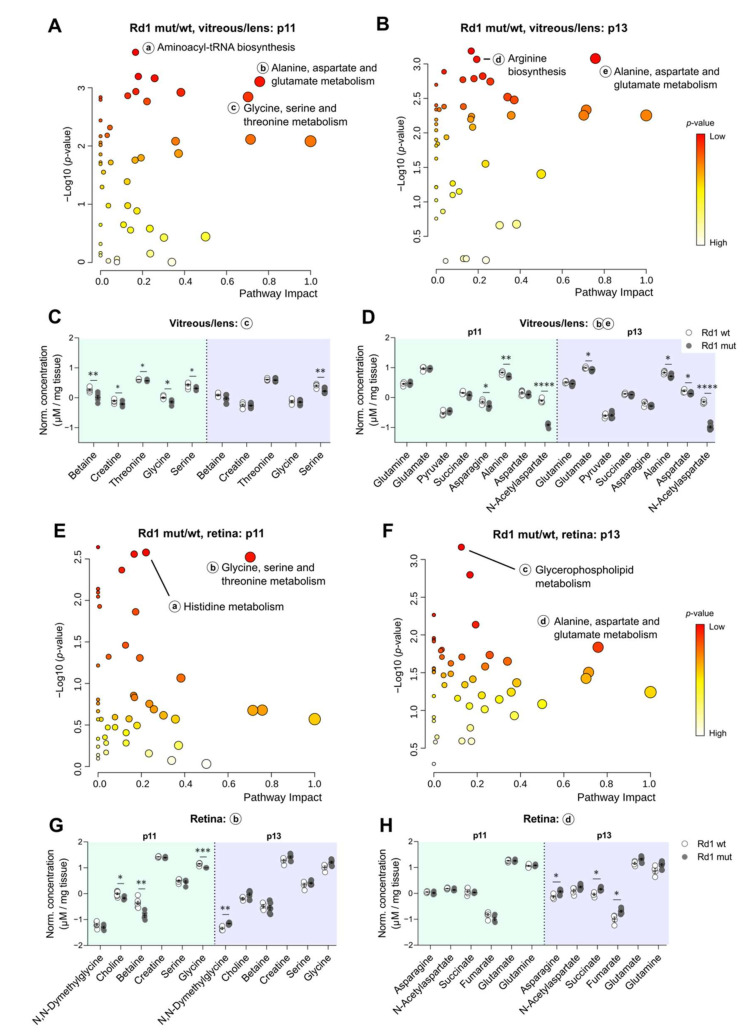
Different metabolic pathways are altered as a result of the degeneration in both eye compartments and at different time-points. (**A**,**B**) Pathway analysis of the metabolites enriched in the vitreous/lens at p11 (**A**) and p13 (**B**) in Rd1 mutant compared to wt mice. Small letters indicate prominent pathways at the specified time-points. (**C**,**D**) Concentration values of metabolites in the vitreous/lens involved in the pathways indicated by the small letter (as in subfigures (**A**,**B**)), at p11 (**C**) and p13 (**D**). (**E**,**F**) Pathway analysis of the metabolites enriched in the retina at p11 (**E**) and p13 (**F**) in Rd1 mutant compared to wt mice. Small letters indicate prominent pathways at the specified time-points. (**G**,**H**) Concentration values of metabolites in the retina involved in the pathways indicated by the small letter (as in subfigures (**E**,**F**)), at p11 (**G**) and p13 (**H**). Rd1 wt, vitreous/lens: *n* = 6 (p11), *n* = 5 (p13); retina: *n* = 7 (p11), *n* = 4 (p13). Rd1 mutant, vitreous/lens: *n* = 6 (p11), *n* = 7 (p13); retina: *n* = 5 (p11), *n* = 6 (p13). Multiple unpaired t test with Holm–Šídák correction for multiple comparisons. * *p* < 0.05, ** *p* < 0.01, *** *p* < 0.001, **** *p* < 0.0001.

Finally, we performed metabolic pathway analysis, focusing first on the vitreous/lens. In this eye compartment, we found “alanine, aspartate, and glutamate metabolism” as the main affected pathway at both analyzed time-points, whereas “glycine, serine, and threonine metabolism” was predominantly affected before eye opening (Figure 6A,B).

A closer look at the metabolites that mediated the enrichment of the term “glycine, serine, and threonine metabolism” confirmed the different levels in Rd1 mutant condition mainly at p11 (Figure 6C), whereas the main metabolites involved in “alanine, aspartate, and glutamate metabolism” appeared differentially represented both at p11 and at p13 (Figure 6D), consistent with the results of the pathway analysis (Figure 6A,B).

Next, we assessed which metabolic pathways were deregulated in the retina. As already observed for the vitreous/lens (Figure 6A), the pathway “glycine, serine, and threonine metabolism” was also the key affected pathway in the p11 retina (Figure 6E). However, the metabolites that led to this enrichment were only partly shared between retina and vitreous/lens. Particularly, Choline, Betaine, and Glycine, which are part of a sub-network taking place mainly in the mitochondria, appeared significantly decreased in the retina of Rd1 mutant mice (Figure 6G), possibly underlying an attempt to promote cell survival via subsequent production of Glutathione or Taurine [40]. Interestingly, at eye opening the main deregulated metabolic pathway was “glycerophospholipid metabolism”, as previously seen also in the Rd1 mutant context alone (Figure 4F), followed by “alanine, aspartate, and glutamate metabolism” (Figure 6F). The latter term was further confirmed by the increase in the levels of Asparagine, Succinate, and Fumarate selectively at this time-point (Figure 6H). Of note, despite the pathway was also enriched in the vitreous/lens at p11 and p13, Asparagine was the only metabolite to be differentially represented in both eye compartments, specifically at p11 in the vitreous/lens (Figure 6D) and at p13 in the retina (Figure 6H).

Taken together, these results highlight changes in the metabolism that are specific to the eye compartment, the time-point and the degenerative state examined, occasionally converging into similar pathways throughout the conditions.

## 3. Discussion

Sight is the sense we mainly rely on to interact with the environment. The visual information crosses the lens and the vitreous before it reaches the retina, a complex neural tissue that detects light stimuli with specialized photoreceptor cells. As such, pathologies affecting the retina, and particularly the photoreceptors, are highly debilitating. Though new mutations causing IRDs are routinely discovered and characterized, the mechanisms eventually leading to progressive retinal degeneration are only known partly. Similarly, mechanisms underlying physiological events, e.g., the consequences of eye opening on the ocular metabolome, are also poorly understood. Here, we chose the mouse as a model to study metabolic changes induced by eye opening in a physiological context as well as in response to degeneration, by providing a comprehensive NMR spectroscopy-based metabolome analysis of wild type and Rd1 retina. To investigate how retinal degeneration affects the levels of metabolites outside the retina, we also analyzed the metabolic changes in the adjacent vitreous/lens compartment. Due to the small size of the mouse eye and the relatively small vitreal volume, we combined vitreous and lens and analyzed them together as vitreous/lens samples.

Using this experimental setting, we have shown that eye opening under physiological conditions seems to be characterized by an overall decrease in the levels of most metabolites in the retina, particularly of metabolites related to purine metabolism (Xanthine; Hypoxanthine; and, albeit not significantly, CMP, Guanosine, and Inosine). While to our knowledge such a drive towards a general reduction of metabolite concentrations has not been previously reported, purine levels have been shown to rapidly decrease in response to light exposure [17]. Interestingly, the results of the metabolic analysis in the vitreous/lens of wt mice did not reveal significant changes between the two analyzed time-points and showed an overall different concentration pattern for all metabolites. This result indicates that the major eye opening-driven metabolic adjustments take place in the retina and confirms that under physiological conditions the exchange of substances between the retina and the two adjacent compartments is tightly regulated [41].

Conversely, in degenerative conditions of the retina this separation can be partially lost [20,33]. Indeed, as we show in the present study in Rd1 mutant mice, selected metabolites (3-Hydroxybutyrate, Sarcosine, and, to a lesser extent, Glucose) displayed similar concentration changes in both eye samples, the retina and the vitreous/lens, before and after eye opening. Both Glucose and 3-Hydroxybutyrate are important energy sources for photoreceptors and are usually provided in a tightly regulated manner by the neighboring retinal pigment epithelial cells, after uptake from the choroidal blood circulation or by cell-intrinsic fatty acid oxidation, respectively [10,12,17,36,42]. In the context of retinal degeneration, these physiological supply mechanisms are compromised and adaptation mechanisms are triggered [39,43,44]. Fatty acid oxidation, which also produces Acetone [11], has been shown to be altered in retinal degeneration [39]. Moreover, the accumulation of 3-Hydroxybutyrate and Acetone as observed in our data could reflect an attempt to compensate for the decrease in Glucose levels at a stage where viable (rod and/or cone) photoreceptors still persists [43,45]. Particularly, the observed increase in 3-Hydroxybutyrate could indicate an imminent switch in retinal and/or photoreceptor metabolism towards fatty acids oxidation to replenish the levels of Acetyl-CoA, a central molecule in many metabolic pathways [46]. Supporting this hypothesis, mice with a retina-specific deletion of the mitochondrial pyruvate carrier 1 (*Mpc1*) display higher consumption of 3-Hydroxybutyrate to compensate for the reduction of Glucose-derived Pyruvate [47]. It is also possible that the accumulation of 3-Hydroxybutyrate and Acetone rather reflects an impairment of the mitochondrial function peaking at eye opening.

Alternatively, 3-Hydroxybutyrate could concur to the antioxidative stress response of photoreceptors [48,49], particularly acting together with Fumarate [45], which we also found increased in the Rd1 mutant retina at eye opening when compared to the wt condition. Oxidative stress is known to play a key role in retinal degeneration and has been the target of several therapeutic approaches [50,51,52,53,54,55,56,57,58]. The exceptional metabolic demand of photoreceptors is paralleled by a combination of different metabolic pathways, including primarily aerobic glycolysis [7,8,9,12], but also oxidative phosphorylation [2,14,15], which when combined with light exposure is one potential source of reactive oxygen species within these cells. Although even in the presence of oxygen more than 80% of all Glucose taken up by photoreceptors is processed via glycolysis [8], the differences we have observed could additionally reflect impairments caused to the oxidative metabolism and further suggest an attempted metabolic adaptation in response to the degeneration.

Indeed, given the additional presence of extramitochondrial respiratory chain enzymes in the rod disk membranes [13,14,15], the loss of outer segments in the initial phases of the degeneration could cause a deficiency of the local source of ATP as well as a potential formation of reactive oxygen species, as previously suggested [59]. The hypothesis of an impaired oxidative mitochondrial and/or extramitochondrial metabolism is further supported by our proteomic data, which show the decrease in the levels of a subunit of the ATP synthase (Atp5f1e) alongside the massive decrease of proteins involved in the visual cascade. With the likely collapse of rod outer segments preceding the actual cellular death, we can speculate that both the loss of the extramitochondrial respiratory chain and the failure of Glucose uptake from the RPE might be the first metabolic impairments to take place and to initiate subsequent metabolic changes eventually concurring to rod cell death [30,31,60].

All nucleotide monophosphate levels appeared increased in Rd1 mutant retinas from p11 to p13, although below the significance threshold, while Hypoxanthine was significantly increased. This observation is in direct contrast with what we observed in wt retinas, thereby proving specific for the degenerative context. We speculate that the higher concentration of metabolites belonging to purine and pyrimidine metabolism reflects the shunt of part of the Glucose to the Pentose Phosphate Pathway (PPP), possibly to counteract the formation of reactive oxygen species [57,59,61], as described in other systems [62]. Thus, the observed decrease of Choline and Betaine at p11 could also support an ongoing attempt to boost the production of antioxidant such as Taurine and Glutathione (GSH), the latter being affected in the Rd1 and Rd10 retinal degeneration models [56,63]. While further studies are required for confirmation, the non-significant increase of Hypotaurine and oxidized Glutathione (GSSG) in our Rd1 mutant vitreous/lens and retina samples, respectively, provides a first hint into this direction.

Finally, by comparing Rd1 mutant and wt conditions in vitreous/lens, we could identify N-Acetylaspartate as a potential novel marker of photoreceptor degeneration. Together with Acetate, whose levels are also significantly decreased at p11, N-Acetylaspartate is synthesized in the mitochondria from Acetyl-CoA. Low levels of N-Acetylaspartate have been associated to neuronal loss and cellular dysfunction [64,65], possibly resulting from a low reservoir of Acetyl-CoA in pathological contexts [66]. Interestingly, a decrease in N-Acetylaspartate was already reported in the vitreous of patients suffering from proliferative diabetic retinopathy, although the authors did not comment on its significance [26]. In the same work, an increase in Arginine was also reported in whole eyes from oxygen-induced retinopathy mice, similarly to what we found in the Rd1 mutant retina compared to wt at eye opening, suggesting converging mechanisms in different models of inherited and acquired retinal degeneration.

Although we believe we shed some light on the metabolic interaction (or lack thereof) between vitreous/lens and retina across time and pathophysiological conditions, we are aware that the presence of the lens and the potential contamination of the retina samples with residual vitreous could affect the described readout. However, as only a small proportion of metabolites show similar significant changes in both compartments, we believe we can exclude any substantial interference that this technical issue could bring. Further analyses with specific metabolic assays are needed to consolidate these data and to understand how these different metabolic pathways concur to determine the rapid loss of the photoreceptors seen in the Rd1 mutant model. Additional time-points, e.g., after p13, could help to further pinpoint the changes taking place specifically at eye opening.

Taken together, we can conclude that under physiological conditions the transition to eye opening in the mouse retina is accompanied by very specific changes in the metabolome, which, however, are not mirrored by metabolic changes in the adjacent vitreous or lens. In the presence of an ongoing photoreceptor degeneration, the retina and vitreous/lens metabolomes are uniquely affected, with only a few metabolites being similarly altered in both compartments. Moreover, such changes are mostly specific for the time-point analyzed, suggesting an additional effect of the developmental stage on the pre-existing degenerative background. Importantly, as an exception to the temporal specificity of these metabolic changes, we propose N-Acetylaspartate as a potential marker of retina degeneration that might be assessed via analysis of vitreous or, possibly, aqueous humor samples from patients.

We envision that our data could pave the way to new lines of investigation and contribute to a better understanding of basic mechanisms underlying retina physiology and pathology.

## 4. Materials and Methods

### 4.1. Animals

Experiments were conducted on *Pde6b^wt^* (Rd1 wt) and *Pde6b^Rd1^* (Rd1 mutant) mice (*Mus musculus*) at postnatal age 11 (p11) and 13 (p13) and with no specific gender bias. The day of birth was considered as p1. All mice were chow-fed and provided with abundance of water, while being kept in a 12 h light/dark cycle. All animal protocols were performed with permission of local authorities (District Government of Upper Bavaria) and in accordance with the German laws on animal welfare (Tierschutzgesetz).

### 4.2. Tissue Isolation

Eyes from Rd1 wt and mutant mice were transferred in a dish with Whatman filter paper and Hank’s Balanced Salt Solution (#14190-144, Thermo Fisher Scientific, Munich, Germany) following enucleation. Subsequently, an excision was carefully performed along the limbus to reveal the lens and the attached vitreous, which were both collected in a tube and directly flash-frozen in liquid nitrogen. With the posterior eye cup exposed, the retina was then gently peeled off the underlying retinal pigmented epithelium and processed as the vitreous/lens. Samples were maintained at −80 °C until further processing.

### 4.3. Protein Extraction and MS Measurements

Approximately 1 mg of retinal tissue was isolated from both Rd1 wt and mutant mice. The tissues were homogenized manually using Micro-homogenizers, PP (Carl Roth GmbH + Co. KG, Karlsruhe, Germany), then further subjected to sonication in a Bioruptor bath sonicator (10 cycles, 30 s ON–30 s OFF) at 4 °C (mainly to shear DNA and get rid of other interfering molecules) after lysing them using the iST Sample Preparation Kit (PreOmics, Martinsried, Germany) according to manufacturer’s instructions. The homogenous lysate was incubated with Trypsin/LysC mixture (supplied with the kit) at 37 °C for two and a half hours. The resulting suspension was centrifuged at 10,000 rpm for 5 min and the supernatant containing the peptides was desalted, purified, and redissolved in 10 µL “Load” solution after drying completely in a speed vac.

For reversed phase HPLC separation of peptides on a Ultimate 3000 nanoLC system (Thermo Fisher Scientific, Munich, Germany), 5 μL of the solution were loaded onto the analytical column (120 mm × 0.075 mm, in house packed with ReprosilC18-AQ, 2.4 μm, Dr. Maisch GmbH, Ammerbuch, Germany), washed for 5 min at 300 nL/min with 3% ACN containing 0.1% FA and subsequently separated applying a linear gradient from 3% ACN to 40% ACN over 50 min. Eluting peptides were ionized in a nanoESI source and on line detected on a QExactive HF mass spectrometer (Thermo Fisher Scientific, Munich, Germany). The mass spectrometer was operated in a TOP10 method in positive ionization mode, detecting eluting peptide ions in the *m*/*z* range from 375 to 1600 and performing MS/MS analysis of up to 10 precursor ions. Peptide ion masses were acquired at a resolution of 60,000 (at 200 *m*/*z*). High-energy collision-induced dissociation (HCD) MS/MS spectra were acquired at a resolution of 15,000 (at 200 *m*/*z*). All mass spectra were internally calibrated to lock masses from ambient siloxanes. Precursors were selected based on their intensity from all signals with a charge state from 2 + to 5 +, isolated in a 2 *m*/*z* window and fragmented using a normalized collision energy of 27%. To prevent repeated fragmentation of the same peptide ion, dynamic exclusion was set to 20 s.

### 4.4. MS Data Processing

Protein identification was performed by MaxQuant 1.5.2.8 software package (Max-Planck-Institute of Biochemistry, Munich, Germany) [67]. Parent ion and fragment mass tolerances were 8 ppm and 0.7 Da, respectively, and allowance for 2 missed cleavages was made. Mouse canonical protein database from Uniprot (release June, 2018), filtered to retain only the reviewed entries was used for the searches. Regular MaxQuant conditions were the following: Peptide FDR, 0.01; Protein FDR, 0.05; Min. peptide Length, 7; Variable modifications, Oxidation (M); Acetyl (Protein N-term); Acetyl (K); Fixed modifications, Carbamidomethyl (C); Peptides for protein quantitation, razor, and unique; Min. peptides, 1; Min. ratio count, 2.

### 4.5. Metabolite Extraction and Spectroscopy

For each group and time-point a total of four to seven samples were used, depending on availability and quality of sampling, and processed as previously described [68]. Briefly, weighed (2–8 mg) frozen retina and vitreous/lens tissues were mixed with 70% aqueous methanol and metabolites were extracted by homogenization in a Bullet Blender 24 (Next Advance Inc, Troy, NY, USA) and centrifugation at 16,000× *g* at 4 °C for 15 min. Extracts were dried under gentle nitrogen stream and reconstituted in 250 μL phosphate buffer (pH 7.4) in deuterated water (D2O) for NMR measurements using a 14.1 T NMR spectrometer. All samples included 0.02 mM of trimethylsilyl-2,3-d4-propionate sodium salt (TSP-d4) as internal standard for quantification.

The collected spectra were processed (phase correction, baseline correction, and referenced to TSP-d4) and the actual concentration of the internal standard in each sample was calculated using a calibrated electronically synthetized digital signal (ERETIC2; Bruker, Billerica, MA, USA). Quantification of absolute concentrations of metabolites was carried out in Chenomx and concentrations were corrected for tissue weight. Three spectra (2 from retina and 1 from vitreous/lens) were excluded from analysis due to insufficient quality.

### 4.6. Analysis of Metabolic Data

The data were analyzed with MetaboAnalyst [69] 4.0 using the generalized logarithm transformed (glog) concentrations. For statistical investigations, principal component analysis (PCA) was performed as unsupervised method to overview all available data. Heatmaps were generated with the Euclidean distance measure and “average” was chosen as clustering method for standardized concentrations (z-scores) of metabolites. For pathway investigation, metabolites were mapped against the *Mus musculus* KEGG database, after being enriched via global Ancova and analyzed in topology according to relative-betweenness centrality. An example of metabolic network selected from the pathway analysis was further plotted in Cytoscape 3.8.2 (Cytoscape Consortium) [70] and processed with MetScape 3.1.3 [71] plugin. For quantitative enrichment analysis, the normalized and scaled (glog) data were compared to the chemical class metabolite sets (sub-class) and displayed as bar-chart view.

### 4.7. Immunohistochemistry

Following eye enucleation, the eyes were punctured with a needle and incubated in 4% paraformaldehyde (PFA) on ice. After 5 min, the anterior part was excised and the vitreous/lens removed. The posterior eye cup was then allowed to fix in 4% PFA on ice for 45 min and later transferred in a 30% sucrose solution at 4 °C. The next day the eye cup was finally embedded in NEG-50 (#6502, Thermo Fisher Scientific, Munich, Germany) and frozen at −80 °C. Coronal cryosections (20 µm) were collected serially at the cryostat and incubated at room temperature for 60 min with a blocking solution consisting of 2% BSA, 0.3% Triton X-100 and PBS. The following primary antibodies were diluted in the same solution and incubated overnight at 4 °C: rabbit anti-iba1 (1:500; #234013, Synaptic Systems, Göttingen, Germany), rat anti-Cd11b (1:500; #101202, BioLegend, Koblenz, Germany), mouse anti-GFAP (1:500; #644701, BioLegend, Koblenz, Germany), sheep anti-cGMP (1:300; courtesy of Jan de Vente, University of Maastricht, The Netherlands). Following several washes in PBS, the sections were incubated for 2 h at room temperature with the following secondary antibodies, all diluted 1:500 in blocking solution: anti-rat Alexa Fluor 488 (#A-21208, Thermo Fisher Scientific, Munich, Germany), anti-rabbit Alexa Fluor 488 (#A-11008, Thermo Fisher Scientific, Munich, Germany), anti-sheep Alexa Fluor 594 (#A-11016, Thermo Fisher Scientific, Munich, Germany), anti-mouse Alexa Fluor 647 (#A-21236, Thermo Fisher Scientific, Munich, Germany), and anti-rabbit Alexa Fluor 647 (#A-32733, Thermo Fisher Scientific, Munich, Germany). The sections were then washed in PBS and counterstained with DAPI (#D1306, Thermo Fisher Scientific, Munich, Germany) prior to mounting (Aqua-Poly/Mount, #18606-5, Hirschberg an der Bergstraße, Germany).

### 4.8. Image Acquisition and Visualization

Images were acquired with an inverted Leica DMI 8 confocal microscope equipped with lasers emitting at wavelengths of 405, 488, 552, and 638, using an objective magnification of 40×. The original images, consisting of multiple z-stacks, were further processed with the open-source software Fiji [72] prior to final elaboration in Inkscape. Plots were generated with GraphPad Prism 9 (GraphPad Software, San Diego, CA, USA).

### 4.9. Statistical Analysis

In order to detect changes in protein levels between Rd1 wt and mutant mice, statistical analysis was performed on the acquired LC-MS/MS data. LIMMA moderated *t*-test [73] was chosen over standard *t*-test as it utilizes full data to shrink the observed sample variance towards a pooled estimate, resulting in far more stable and powerful inference compared to ordinary *t*-test. Missing values in the data were imputed from randomly drawn values from a distribution meant to simulate values below the detection limit of the MS instrument [74]. An in-house built R-package [75] was used for analyzing the proteomic data. Proteins detected in at least one of the biological replicates and having at least > 1% sequence covered were considered for statistical analysis.

Gene ontology analysis was performed using the Functional annotation tool from DAVID Bioinformatics Resources 6.8, NIAID/NIH (Laboratory of Human Retrovirology and Immunoinformatics, Frederick, USA) [76,77].

The entire list of enriched proteins in either Rd1 wt or mutant was used as input against a background that contained the entire proteome from *Mus musculus*. Furthermore, the biological processes were ranked according to “Enrichment score” defined as follows: Enrichment score = (Fold enrichment/*p*-value) × Gene count. All the variables are as they are defined in DAVID. Genes involved in metabolic processes (as defined in Uniprot: metabolic process (0008152)) were identified in the entire enriched list of proteins and Log2 (Fold change) of those proteins (from LIMMA analysis) was used to assess the overall changes in tissue metabolism.

For Volcano plots of metabolites, all metabolome data were analyzed in Perseus [74] via a two-sample Student’s t-test, followed by Benjamini–Hochberg false discovery rate (FDR) test. The value of the FDR was set on 0.1 for all experiments and the Log2 (fold change) cut-off was set on 1. Data points appearing in gray indicate values that did not pass both the statistical and the cut-off threshold. Data points in black indicate statistically significant values that did not pass the cut-off threshold.

Statistical analysis of selected metabolites was performed with GraphPad Prism 9 (GraphPad Software, San Diego, CA, USA) via unpaired t test with Holm-Šídák correction for multiple comparisons, setting *p* < 0.05 as threshold for significance.

## Data Availability

The data that support the findings of this study are available from the corresponding author upon reasonable request.

## Supplementary Materials

Supplementary materials can be found at https://www.mdpi.com/1422-0067/22/5/2345/s1.

## Abbreviations

IRDs	Inherited retinal disorders
RPE	Retinal pigmented epithelium
Rd1	*Pde6b^Rd1^* mutation
cGMP	Cyclic guanosine monophosphate
PCA	Principal component analysis
MSEA	Metabolite set enrichment analysis
AMP	Cyclic adenosine monophosphate
CMP	Cyclic cytidine monophosphate
PPP	Pentose phosphate pathway
GSH	Reduced Glutathione
GSSG	Oxidized Glutathione
GSEA	Gene set enrichment analysis
